# Belatacept-based immunosuppression in heart transplant recipients: A single center experience

**DOI:** 10.1016/j.jhlto.2025.100277

**Published:** 2025-05-22

**Authors:** Wairimu Magua, Maggie Wang, Darlington Pobee, Anna Morris, Alexis K. Okoh, Emily M. Eichenberger, Geeta M. Karadkhele, Divya Gupta, J. David Vega, Christian P. Larsen, Anne Van Beuningen, Alanna A. Morris

**Affiliations:** a Department of Surgery, Transplant, Emory University, Atlanta, GA; b Department of Medicine, Cardiology, Emory University, Atlanta, GA; c Department of Pathology and Laboratory Medicine, Emory University, Atlanta, GA; d Department of Medicine, Infectious Diseases, Emory University, Atlanta, GA

**Keywords:** Belatacept, De novo donor specific antibodies, DSA, Antibody mediated rejection, AMR, Calcineurin inhibitors, Renal function

## Abstract

**Background:**

We aim to describe the effect of belatacept on de novo donor-specific antibodies (DSA) formation, rejection, and renal function in heart transplant recipients.

**Methods:**

The cohort comprises 60 adult heart or heart-kidney recipients transplanted between 2005 and 2022. Twelve recipients initialized at ∼90 days post-transplantation on a belatacept-based immunosuppression regimen with tapered tacrolimus trough levels were matched to 48 standard tacrolimus-based regimen controls. Differences in the distribution of recipients with emergent de novo DSA and rejection were assessed over the first 85 days baseline period, the average duration pre-belatacept. Survival analysis assessed regimen group differences in the probability of remaining de novo DSA and rejection free over the follow-up period spanning 86 to 540 days. Renal function and cytomegalovirus viremia were examined as secondary outcomes.

**Results:**

There were no statistically significant regimen group differences in the distribution of recipients with de novo DSA or rejection during the baseline period. Furthermore, differences in the probability of remaining de novo DSA and rejection free during the follow-up period remained insignificant (log-rank test, *p* = 0.12). Belatacept-treated recipients, at follow-up, had no incidence of developing de novo DSA, unlike 19% of the controls. Additionally, there were no statistically significant differences in acute cellular and antibody mediated rejection events, renal function, and CMV viremia by regimen group.

**Conclusion:**

Recipients treated with belatacept-based regimen exhibited a trend of reduced de novo DSA development compared to standard tacrolimus-based regimen controls. Larger studies are needed to evaluate the benefit of belatacept use in heart transplant populations.

## Background

The formation of de novo donor-specific antibodies (DSA) following heart transplant (HT) and heart-kidney transplant (HKT) has been associated with antibody-mediated rejection (AMR), cardiac allograft vasculopathy, transplant glomerulopathy, and increased mortality.^1^, ^2^, ^3^, ^4^, ^5^ Notably, de novo anti-human leukocyte antigens (HLA) DQ and DR DSA are associated with increased frequency of de novo DSA and immunogenicity, resulting in poorer outcomes compared to anti-HLA DP and class I DSA.^2^, ^3^, ^6^, ^7^, ^8^, ^9^ The incidence among HT recipients of developing de novo DSA is clinically significant, with observed rates ranging between 10% and 30%.^3^, ^10^, ^11^ Knowledge on the risk factors that contribute to the development of de novo DSA in HT recipients remains scarce.^12^, ^13^ The impact of immunosuppression intensity has been shown to be a risk factor for developing de novo DSA in other solid organ transplant populations.^14^ Non-adherence to immunosuppression regimens also confers increased risk of developing de novo DSA.^15^ Immunosuppression regimens based on calcineurin inhibitors (CNI) are the standard maintenance approach following heart transplantation.^16^ The routine use of CNI is often complicated by nephrotoxicity and adverse cardiovascular side-effects such as hypertension and hyperlipidemia and by the new-onset diabetes^17^, ^18^ further contributing to increased morbidity and mortality in this population. Therefore, there remains a need for improved immunosuppression regimens that will prevent the formation of de novo DSA and graft rejection without the burden of nephrotoxicity and/or increased cardiometabolic risk.

Belatacept is a recombinant soluble fusion protein that selectively inhibits T-cell activation through co-stimulation blockade.^19^ Belatacept was approved by the Food and Drug Administration (FDA) in 2011 as an alternative immunosuppressant to CNI in kidney transplant recipients after several clinical trials demonstrated its efficacy in preventing allograft rejection without the side-effect of nephrotoxicity,^20^, ^21^, ^22^ and showed improved lipid profiles and glycemic control.^23^, ^24^ However, such large scale registrational trials to evaluate belatacept as an alternative immunosuppression agent in HT populations have not yet been undertaken.

Among kidney transplant populations, belatacept has been shown to constrain pre-existing DSA post-transplant^25^ and confer lower incidence of de novo DSA.^26^ Emerging evidence suggests that belatacept, when combined with proteosome inhibitor-based desensitization, can significantly attenuate pre-existing HLA class I and II antibodies in highly sensitized HT candidates and can subsequently increase the likelihood of matching the transplant candidates with donors.^27^, ^28^ Furthermore, HT recipients with known renal dysfunction have been shown to exhibit improved kidney function following conversion from CNI-to belatacept-based immunosuppression.^29^

We developed a belatacept-based regimen protocol that includes tacrolimus tapering dosing trough levels, guided by our previous findings on the relatively high risk of accelerated graft failure due to Class II de novo DSA in our HT population and by extant evidence on the role of belatacept in attenuating development of de novo DSA in renal transplant recipients.^3^, ^25^ In this study, we examine whether belatacept-based immunosuppression mitigates the development of de novo DSA in HT or HKT recipients who were initialized early (∼90 days) on a belatacept-based regimen. We describe observed rejection and renal function before and after belatacept initialization. We hypothesize that HT and HKT recipients on a belatacept-based immunosuppression regimen will have lower incidence of de novo DSA development.

## Methods

### Study population

This is a retrospective observational study of adult first HT or dual HKT transplant recipients who were transplanted between 2005 and 2022 and treated with either belatacept-based or tacrolimus-based standard immunosuppression regimen following transplantation at Emory Transplant Center (ETC). All recipients transplanted from 2020 onward were assessed for their eligibility to receive belatacept-based immunosuppression regimen. Since belatacept lacks FDA approval in the HT population, insurance companies commonly withhold coverage for the use of belatacept. Belatacept-based regimen by protocol was initialized approximately 90 days after transplantation. Eligibility criteria required recipients to have a negative Epstein-Barr virus (EBV) serostatus to mitigate the risk of lymphoproliferative disorder that has been associated with belatacept.^30^ Additionally, the recipients were required to have either cytomegalovirus (CMV) moderate-risk (Donor/Recipient: D+/R+ or D-/R+) or CMV low-risk (D-/R-) serostatus profiles given the known and sustained defects in viral load control in belatacept-treated recipients with a high-risk CMV serostatus profile.^31^ Recipients with any history of post-transplant lymphoproliferative disorder, lymphoma, or hematologic malignancy were also ineligible for belatacept-based regimen. The tacrolimus-based regimen group was selected from eligible belatacept naïve recipients who received their first HT or HKT and who had similar EBV and CMV serostatus risk profiles as recipients in the belatacept-based regimen group. All belatacept-treated recipients self-identified as either Black or White. Since extant literature suggests that Black recipients have a higher risk of antibody mediated rejection,^32^ recipient race was included as a matching criterion.

Belatacept recipients were matched 1 to 4 to a historical control group exactly by race, gender, and type of transplant (HT or HKT) using a genetic algorithm and approximately by age using Mahalanobis distance as implemented by the *MatchIt R* package.^33^ The quality of matches was evaluated using standardized mean differences of the matched characteristics between the recipient groups. The first 85 days following transplantation were designated as the baseline period, reflecting the average duration before initialization of the belatacept-based regimen. Days 86 to 540 (18 months) were designated as the follow-up period.

### Study outcomes

The primary outcome was the estimated probability of remaining free from developing de novo DSA during the follow-up period and up to 18 months. The secondary outcomes included: 1) preservation of renal function, 2) AMR, 3) acute cellular rejection (ACR), and 4) development of CMV viremia.

### Immunosuppression

The belatacept-based and tacrolimus-based immunosuppression recipient groups received a basiliximab induction (20 mg IV) during the intraoperative period and at day 4 following transplantation. Corticosteroids were also administered during the intraoperative period and weaned off at 12 months. Mycophenolate mofetil (MMF) maintenance of 1,000 mg was administered twice a day and adjusted based on recipient clinical needs. Recipients in the tacrolimus-based immunosuppression group received tacrolimus dosing target trough levels of 8-12 ng/ml at 0-3 months, 6-10 ng/ml at >3-6 months, and 5-8 ng/ml at 6 months onward.

Recipients in the belatacept-based immunosuppression group received tacrolimus tapering dosing target trough levels of 8-12 ng/ml at 0-3 months, 5-8 ng/ml at >3-6 months, and 3-5 ng/ml at >6-18 months. Tacrolimus continuation or trough goal was evaluated after 18 months. Belatacept dosing was adapted from the ETC kidney transplant protocols, which have been used in over 2,000 patients. The belatacept intravenous infusion was initiated at a dose of 5 mg/kg at ∼90 days and maintained monthly within +/- 5 day time window. The infusion dose by protocol was assessed annually for adjustment. Infusions during the first 12 months were administered at ETC and optionally in other outpatient clinic settings thereafter at a standard dose determined at the time of transition.

### HLA testing

Mismatches were discerned by evaluating the antigen phenotypes of donors and recipients. Presence of Class I and II HLA antibodies in each patient case were identified using FlowPRA Class I and II Screening Tests (One Lambda, Inc., Canoga Park, CA) and LABScreenTM Single Antigen HLA Class I and LABScreen^TM^ Single Antigen HLA Class II kits (One Lambda, Inc/Thermofisher). Details of methodologies used are included in the Supplemental Material. cPRA was calculated for each case (https://optn.transplant.hrsa.gov/data/allocation-calculators/cpra-calculator/) for any specificity identified according to the MFI thresholds for positivity at the time of transplantation, regardless of whether it was made an unacceptable antigen for organ allocation, indicating the overall HLA sensitization in the patient. Following transplantation, recipients were assessed for de novo DSA at 2 weeks and at 1, 2, 3, 6, 12, and 18 months via single antigen bead analysis as stated above.

Flow cytometric crossmatches were performed by incubating recipient sera with pronase-treated surrogate donor peripheral blood lymphocytes. Bound IgG was detected via a secondary FITC-conjugated, Fc-specific, anti-human IgG antibody (Jackson Immunoresearch). Shifts in fluorescence intensity were assessed in ΔMESF (molecules of equivalent soluble fluorochrome) using Quantum MESF Kit (Bangs Laboratories). All sera are run in duplicate along with positive and negative control sera. Further details of methodologies used are included in the Supplemental Material.

### Renal function

Estimated glomerular filtration rate was calculated using Chronic Kidney Disease Epidemiology Collaboration race free 2021 equation^34^ using the *nephro*^35^ R package.

### Rejection

Rejection protocols have evolved over the study transplant date timeline. Aligned with the current protocol, standard rejection surveillance was followed with all rejection events identified using biopsies during the first 12 months following transplantation. Thereafter, all rejection events were considered to be treated-rejection. Diagnosis of ACR or AMR were made based on endomyocardial biopsies. The C4d immunohistochemical method was applied to stain the biopsies, with C4d staining categorized as positive when it exhibited either widespread staining or focal involvement in more than 50% of capillaries. ACR and AMR were defined based on 2004 International Society for Heart and Lung Transplantation guidelines^36^ and pathologic diagnosis formulation respectively.^37^

### CMV prophylaxis and viremia

Recipients with moderate-risk CMV serostatus profiles (D-/R+ or D+/R+) received prophylaxis with valganciclovir starting at day 21 post-transplantation for 3 months. Recipients with low-risk CMV serostatus profiles (D-/R-) received HSV prophylaxis with valacyclovir starting on day 21 post-transplantation for 3 months. The dosing of CMV prophylaxis was adjusted in recipients with renal impairment. CMV viremia was classified into three groups: undetectable or low viral loads [0, 500) copies/ml, moderate viremia with [500, 10^4^) copies/ml; and severe viremia (viral load >10^4^) copies/ml.

### Analytic methods

The cohort clinical and demographic baseline characteristics were examined. Continuous and categorical variables were described using a combination of counts, percentages, medians with interquartile ranges, and means with standard deviations. DSA were classified as de novo when they first appeared at any point during the baseline or follow-up time period. Over the baseline period, regimen group differences in the distribution of recipients who formed de novo DSA and who experienced ACR and AMR were described. Kaplan-Meier survival curves were used to examine the probability of remaining free from developing de novo DSA, AMR, and ACR during the follow-up period and up to 18 months following transplantation. The log-rank test was used to quantify the survival curve differences observed. Additionally, the percent distribution of recipients across different levels of CMV viremia severity by regimen groups during the follow-up period was examined.

Statistical comparisons between the groups were assessed using *t*-tests, Wilcoxon signed-rank tests, chi-squared, or Fisher’s exact test as appropriate. Significant differences in distribution between the two groups were reported with *p*-values at significance threshold of *p*-value ≤0.05. Statistical analyses were conducted using R (version 4.2.2)^38^ in RStudio (version 2023.9.1.494).^39^ Analyses and visualization were completed using *MatchIt,*^33^ *tidyverse,*^40^ *survival,*^41^ and *survminer,*^42^ *gtsummary,*^43^ and *ggplot*^40^ R packages.

## Results

### Study cohort

**Figure 1:**
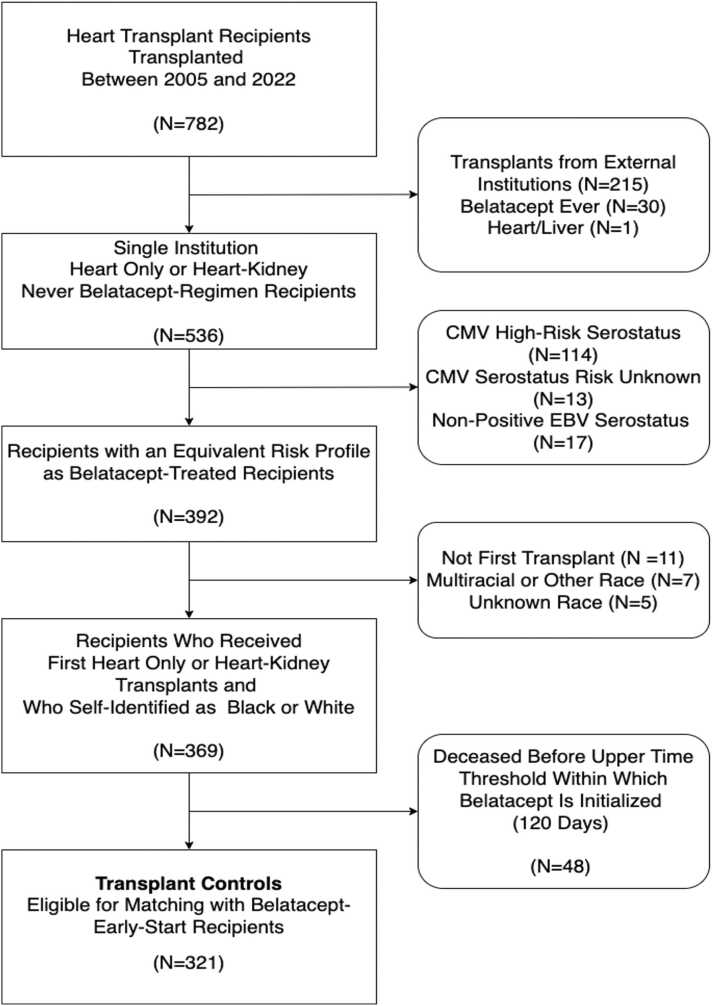
Flow diagram showing selection criteria for recipients treated with standard tacrolimus-based immunosuppression regimen, leading to the identification of those eligible for selection as matched controls.

Out of the 782 HT recipients who underwent transplantation between 2005 and 2022, approximately half (*N* = 392) were belatacept naïve HT or HKT transplant recipients who were transplanted at ETC and who had similar CMV and EBV serological risk profiles as those exposed to belatacept-based regimen (Figure 1). Out of the 392 recipients, a total of 321 were eligible as potential matched controls, as they had no prior transplants, could be identically matched by race to those in the belatacept-based immunosuppression group, and had survived to at least 120 days—the upper time threshold within which belatacept was initialized. The 1 to 4 matched groups, with exact matching on variables race, gender, and type of transplantation and approximate matching by age, yielded 48 standard tacrolimus-based immunosuppression control recipients. The standardized mean difference in age between the regimen groups was 0.093 and with a variance ratio of 0.95, indicating a negligible age difference between the two groups (Supplementary Figure S1).

There were no significant differences in baseline characteristics between the recipients in the belatacept-based and tacrolimus-based regimen groups (Table 1). The recipient median age was 49 years. At least two-thirds of the recipients were Black males. There were no significant differences in the distribution of recipients by type of transplantation, primary diagnosis, calculated panel reactive antibodies, crossmatch positivity, primary diagnosis, comorbidities, serological risk profiles, and kidney function. Body mass index was similarly elevated among recipients in the belatacept- and tacrolimus-based immunosuppression regimen groups, respectively. Recipients in both groups equivalently utilized pre-transplant mechanical circulatory support and had similar post-transplant length of hospital stay.

**Table 1:** Baseline Characteristics of Belatacept and Standard Immunosuppression Regimen Groups Transplanted Between 2005 and 2022

Description	Total	Overall *N* = 60^a^	Immunosuppression Regimen		*p*-value^b^
			Standard *N* = 48^a^	Belatacept *N* = 12^a^	
*Age at Transplant*	60	49 (40, 55)	49 (40, 55)	48 (41, 52)	0.73
*Race*	60				>0.99
Black		45 (75%)	36 (75%)	9 (75%)	
White		15 (25%)	12 (25%)	3 (25%)	
*Sex*	60				>0.99
Female		5 (8%)	4 (8%)	1 (8%)	
Male		55 (92%)	44 (92%)	11 (92%)	
*Type of Transplant*	60				>0.99
Heart/Kidney		10 (17%)	8 (17%)	2 (17%)	
Heart		50 (83%)	40 (83%)	10 (83%)	
*BMI*	60	28 (24, 30)	27 (24, 30)	30 (24, 33)	0.34
*Primary Diagnosis*	60				0.22
Congenital Heart Disease		5 (8.3%)	3 (6.3%)	2 (17%)	
Ischemic Cardiomyopathy		12 (20%)	11 (23%)	1 (8.3%)	
Non-Ischemic Cardiomyopathy		43 (72%)	34 (71%)	9 (75%)	
*Hyperlipidemia*	60	19 (32%)	14 (29%)	5 (42%)	0.49
*Diabetes Mellitus*	60	16 (27%)	11 (23%)	5 (42%)	0.27
*Pre-Tx Durable MCS*	60	15 (25%)	11 (23%)	4 (33%)	0.47
*BaselineEstimated GFR*	60	52 (39, 72)	52 (38, 70)	56 (41, 77)	0.58
*CMV Serostatus Risk*	60				0.40
Low		10 (17%)	7 (15%)	3 (25%)	
Moderate		50 (83%)	41 (85%)	9 (75%)	
*Donor Age*	59	25 (21, 30)	25 (21, 32)	28 (23, 28)	0.96
Unknown		1	1	0	
*Donor Sex*	60				0.57
Female		5 (8.3%)	5 (10%)	0 (0%)	
Male		55 (92%)	43 (90%)	12 (100%)	
*Calculated PRA*	60				0.75
0-20		54 (90%)	43 (90%)	11 (92%)	
21-40		3 (5%)	3 (6.3%)	0 (0%)	
41-60		3 (5%)	2 (4.2%)	1 (8.3%)	
*Crossmatch Positivity*					
T-cell	60	0 (0%)	0 (0%)	0 (0%)	
B-cell	60	11 (18%)	8 (17%)	3 (25%)	0.68
B-cell attributable to DSA	60	2 (3.3%)	1 (2.1%)	1 (8.3%)	0.36
*Tx Length of Stay*	60	15 (11, 22)	14 (11, 22)	16 (10, 21)	0.98

Recipient race is self-reported. The classification of race aligns with that used by the United Network for Organ Sharing. Abbreviations: BMI, Body mass index; Tx, Transplant; MCS, Mechanical circulatory support; GFR, Glomerular filtration rate; CMV, Cytomegalovirus; PRA, Panel reactive antibody.

a Median (IQR) or Frequency (%).

b Wilcoxon rank sum exact test; Fisher’s exact test.

### Trends in De Novo DSA Formation

**Figure 2:**
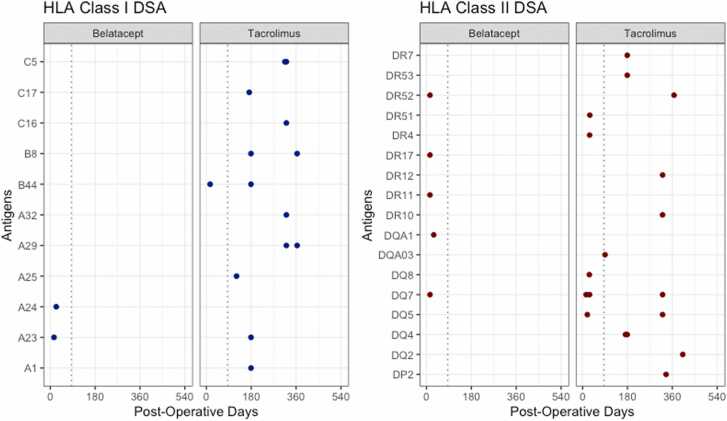
The blue dotted lines separate two periods. The baseline period, in which antigens against HLA class I or II DSA developed within the first 85 days in both immunosuppression groups, and the follow-up period.

All recipients survived to 120 days, the upper time threshold within which belatacept-based immunosuppression was initialized. There were no statistically significant differences by regimen groups in the distribution of recipients who developed class I and II de novo DSAs during the baseline period (Table 2). During the baseline period, class II DSA accounted for most of the DSA formation in both belatacept-based and tacrolimus-based regimen groups (Figure 2) and, by the end of the follow-up period, all de novo DSA that had formed during the baseline period had MFI values were between 500 and 6,000 in the belatacept-treated recipient group and between 2,000 and 12,000 in the tacrolimus-treated recipient group.

**Table 2:** Distribution of Recipients Who Developed De Novo DSA by Immunosuppression Regimen and by Antigen Class During the Baseline Period, Within 85 Days, the Mean Number of Days Before the Initialization of Belatacept-Based Immunosuppression Regimen

Human Leukocyte Antigens	Tacrolimus Regimen *N* = 48^a^	Belatacept Regimen *N* = 12^a^	*p*-value^b^
Class			0.09
No DSA	42 (88%)	8 (68%)	
Class I DSA	1 (2%)	2 (16%)	
Class II DSA	5 (10%)	2 (16%)	

HLA Class I loci include HLA-A, HLA-B, and HLA-C.

HLA Class II loci include HLA-DR, HLA-DQ, and HLA-DP.

a *n* (%).

b Fisher’s exact test.

**Figure 3:**
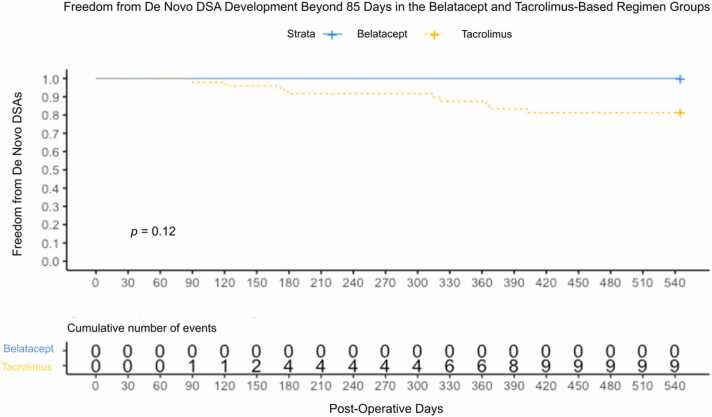
Freedom from developing de novo DSAs in the belatacept- and tacrolimus-based immunosuppression regimen groups during the follow-up period. The suvival curves are anchored at day 85 post-transplanation. Only events occurring thereafter (during the follow-up period) are included in the analysis.

During the follow-up period, no class I or class II de novo DSAs were detected in the belatacept-based regimen group (Figure 2, Figure 3). In contrast, ∼19% of the recipients in the tacrolimus-based regimen group developed de novo DSAs during the follow-up period, with most antibodies targeting HLA class II antigens. Although differences in the probability of remaining free from de novo DSA formation during follow-up was not statistically significant by regimen group, tacrolimus-treated recipients exhibited a trend of increased de novo DSA development. Given hazard ratios are calculated based on events in each group, having no events among the belatacept-based regimen recipients implies that model estimated hazard ratios are undefined.

### No differences in rejection, CMV viremia, and renal function

**Figure 4:**
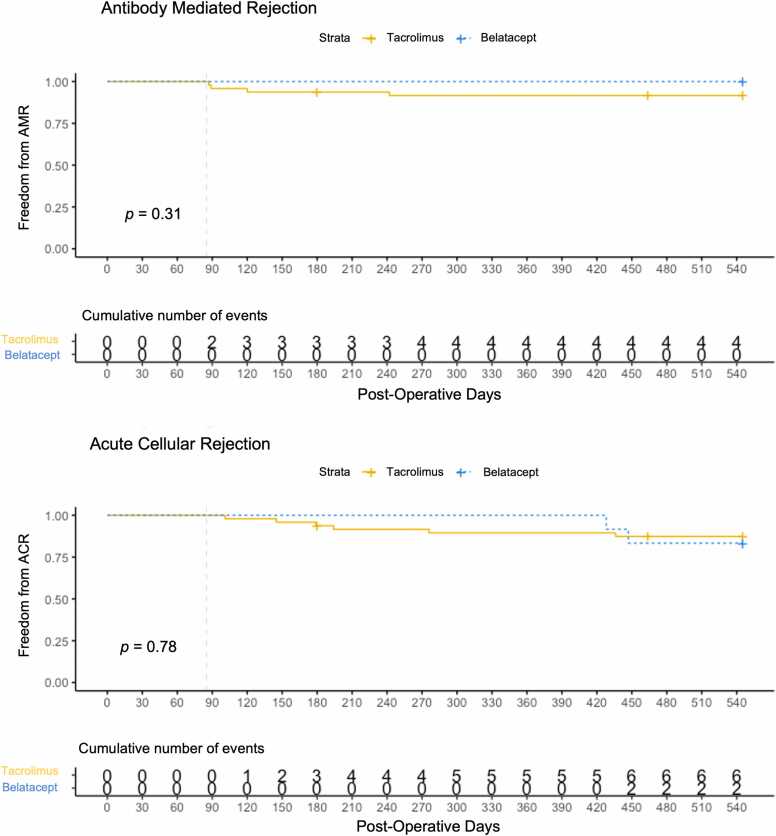
Freedom from developing antibody mediated and acute cellular rejection by regimen groups. The survival curves are anchored at day 85 post-transplanation. Only events occuring thereafter (during the follow-up period) are included in the anaysis. (**A**) Three recipients had histopathologic AMR alone (negative C4d/IF) or immunopathologic AMR alone (+C4d/IF) and 1 recipient had severe pathologic AMR. (**B**) All eight ACR rejection were mild or low grade.

There were no statistically significant differences between the recipient groups in the development of AMR and ACR during the baseline line period (Table 3). Furthermore, there were no significant differences between the belatacept- and tacrolimus-based regimen groups in freedom from AMR (log-rank test: *p-value* = 0.31) and ACR (log-rank test *p-value* = 0.78) during the follow-up period (Figure 4). It is notable that no AMR events occurred in the belatacept-based regimen group compared to the tacrolimus-based regimen groups (0% versus 8%, Supplemental Table S1). All cases of ACR were characterized as mild. There were also no significant differences in renal function or in the development of CMV viremia at 18 months between belatacept-based and tacrolimus-based immunosuppression groups (Table 4). Similarly, the actual tacrolimus trough levels showed similar trends in both regimen groups (Figure S2). Two recipients deceased—one at 6 months and the other at 15 months in the tacrolimus-treated recipient group. One recipient discontinued the belatacept-based regimen at month 8 due to parvovirus and subsequently resumed the belatacept-based regimen at month 13. A second recipient discontinued belatacept-based regimen due to non-adherence. No recipients in the belatacept-treated recipient group became deceased.

**Table 3:** Rejection Events During the Baseline Period, Within 85 Days, the Mean Number of Days Before the Initialization of Belatacept-Based Immunosuppression Regimen

Events During Pre-Belatacept Period	Tacrolimus Regimen *N* = 48^a^	Belatacept Regimen *N* = 12^a^	*p*-value^b^
*AMR, Antibody Mediated Rejection*			**0.5**
pAMR0	36 (75%)	11 (92%)	
pAMR1	7 (15%)	0 (0%)	
pAMR3	5 (10%)	1 (8%)	
*ACR, Acute Cellular Rejection*			**0.15**
0R	31 (65%)	8 (67%)	
1R	13 (27%)	1 (8%)	
2R	4 (8%)	3 (25%)	

**AMR:** pAMR0, no histopathologic or immunopathologic features; pAMR1, presence of either histopathologic or immunopathologic features; pAMR3, presence of severe histopathologic and immunopathologic features.

**ACR:** Grade 0R (no rejection); Grade 1R (mild), interstitial and/or perivascular infiltrate with up to 1 focus of myocyte damage; Grade 2R (moderate), two or more foci of infiltrate with associated myocyte damage.

a *n* (%).

b Fisher’s exact test.

**Table 4:** Renal Function and CMV Viremia Status as Secondary Outcomes Evaluated by Immunosuppression Regimen Group Over the Follow-up Period, 86 to 540 Days Following Transplantation

Description	Total	Overall *N* = 60^a^	Immunosuppression Regimen		*p*-value^b^
			Tacrolimus *N* = 48^a^	Belatacept *N* = 12^a^	
*Estimated GFR*					
*Months, Post-Transplant*					
12 Months	59	61 (52, 74)	61 (53, 72)	61 (50, 79)	**0.95**
Unknown^**3**^		1	1	0	
18 Months	51	63 (53, 71)	62 (53, 70)	66 (61, 80)	**0.30**
Unknown^**3**^		9	6	3	
*CMV Viremia Status*	**58**				**>0.99**
*18 Months, Post-Transplant*					
No Viremia		55 (95%)	43 (93%)	12 (100%)	
Moderate Viremia		2 (3.4%)	2 (4.3%)	0 (0%)	
Severe Viremia		1 (1.7%)	1 (2.2%)	0 (0%)	
Unknown^3^		2	2	0	

a Median (IQR) or Frequency (%).

b Fisher’s exact test (inverse probability weights account for missing observations). Cytomegalovirus (CMV): undetectable or low viral loads [0, 500) copies/ml, moderate viremia [500, 10^4^) copies/ml; and severe viremia (viral load >10^4^) copies/ml. Included in the unknown group, one recipient deceased at 6 months and the other at 15 months.

## Discussion

We report the use of belatacept-based immunosuppression regimen in 12 HT and HKT adult recipients, transplanted between 2005 and 2022, who were initialized early (∼85 days) to a belatacept-based regimen, with the follow-up spanning 86 days to 18 months following transplantation. The belatacept- and tacrolimus-based immunosuppression regimen groups were compared for the freedom from de novo DSAs during follow-up and up to 18 months. Recipients on the belatacept-based regimen during follow-up showed a trend of no incidence of de novo DSA compared to the tacrolimus-based immunosuppression controls. Although the differences between the groups emerged as not statistically significant, the comparability in the baseline clinical and demographic characteristics and in the proportion of recipients who formed DSA during the baseline period suggests further investigations are warranted for new insights into the potential benefits that belatacept may confer to HT and HKT recipients. When compared to recipients in the tacrolimus-based regimen group, those in the belatacept-based regimen group remained free from AMR events during the follow-up period, although both AMR and ACR events, statistically, did not differ significantly by group. The analysis also showed no renal function advantage in the belatacept-based regimen group over the tacrolimus-based regimen group. Consistent with extant evidence, the introduction of belatacept-based immunosuppression regimen in low-risk and moderate-risk CMV serostatus recipients showed similar incidence of CMV viremia to that in the tacrolimus-based regimen recipient group.^31^, ^44^

The early use (≤3 months) of belatacept as a primary immunosuppression agent in HT has been described by Lauany et al.^29^ While the belatacept-treated recipient population mirrored ours in size, a key distinction from our study was their focus on the potential use of belatacept as a rescue agent from nephrotoxicity conferred by CNI regimens in recipients with transient renal failure. Launay et al. reported significant improvement in renal function. While this result is aligned with those of kidney transplant recipients,^21^ the deviation from our findings may be partially attributed to their focus on recipients with transient renal failure. However, important knowledge gaps remain regarding the renal sparing benefit that optimized belatacept-based protocols may have in the general HT populations and the impact such protocols may have on attenuating the risk acute rejection.^45^ Nonetheless, this study and that of Lauany et al. indicate a favorable belatacept safety profile.

The trend of restrained de novo DSA among HT recipients in the belatacept group is worth noting for several reasons. De novo DSA are formed in 10%-30% of the HT population.^1^, ^2^, ^3^, ^6^, ^11^ Donor specific antibodies against HLA DQ and DR antigens have been implicated in the development of antibody mediated rejection and decreased allograft survival among other outcomes in HT recipients.^6^, ^12^, ^14^, ^34^, ^46^, ^47^ In particular, DSA developed against HLA DQ antigens have been found to be more immunogenic than those developed against other HLA antigens. The presence of de novo DSA is a salient risk factor for the development of AMR followed by related adverse effects, including graft dysfunction, coronary allograft vasculopathy, and graft failure.^46^

While belatacept has not been approved for use in heart-transplant recipients, there is growing interest in the potential to leveraging belatacept-based regimens in this population as it been shown to confer a lower risk of developing de novo DSA and to constrain existing de novo DSA among kidney transplant recipients.^25^, ^26^, ^48^ The attenuated development of de novo DSA is attributed to belatacept’s inhibition of the CD28-CD80/86 signaling pathway, which is crucial for T-cell activation. This pathway inhibition reduces the likelihood of B-cell differentiation into antibody-producing plasma cells, resulting in the decreased incidence of de novo DSA.^46^, ^49^ Furthermore, belatacept, in the absence of CNI-based immunosuppressive agents, has been shown to confer better lipid profile and to bypass the nephrotoxic and cardiometabolic adverse effects resulting from long term use of CNIs.^20^, ^21^, ^24^, ^50^ However, the extent to which such gains can be realized in the HT and HTK transplant populations using belatacept-based regimens optimized with tapered CNI immunosuppressive agents should be further examined in clinical trials or larger observational studies with longer follow-up periods.^45^

This is a retrospective small study conducted in a single institution, and hence it is limited in scope. Although the researchers extracting donor-specific antibody data were not blinded, a separate blinded reviewer validated the extracted data, resolved ambiguities, and interpreted the results. This helped mitigate some potential data extraction bias. Despite growing literature on the potential benefit of using belatacept-based regimens in HT recipients, commercial payers often deny immunosuppression coverage because belatacept lacks FDA approval for this population. Furthermore, insurance approval was limited to private payers, potentially introducing selection bias. Consequently, the sample size of belatacept-treated recipients remained restricted. Additionally, rejection protocols have evolved over the course of the study's transplant timeline, which may introduce limitations in interpreting rejection events. Given this is a treatment comparison study rather than a traditional case-control, the focus in matching was to achieve balance in key covariate confounders. Despite its limitations, this study generated preliminary data that can provide valuable insights for future sample size estimation. In particular, covariate standardized mean differences between the immunosuppression groups can serve as standardized effect sizes that can help refine estimated minimum detectable effects for future sample size requirements. These preliminary data can guide future large-scale multi-institutional randomized clinical trials or observational studies of HT recipients with longer-term follow-ups to determine the sustained effects of a belatacept-based regimen on attenuated de novo DSA formation and rejection and on renal function rescue.

## Author Contributions

Authors WM, CPL, AAM were responsible for study concept and design. DP, MW, GMK contributed to data collection. WM conducted data analysis. WM, MW, DP led in drafting of the manuscript. WM, MW, DP, AM, AKO, EME, GMK, DG, DV, CPL, AVB, AAM significantly contributed to data interpretation and critical review of manuscript and approved the article, The study funding was secured by WM and CPL.

## Disclosure Statement

The authors of this manuscript have conflicts of interest to disclose as described by The Journal of Heart and Lung Transplantation. CPL is an Advisor to Eledon, CareDx and Bristol-Myers Squibb. WM, MW, DP, AM, AKO, EME, GMK, DG, JDV, AVB, and AAM have no conflicts of interest to disclose.

## Declaration of Competing Interest

The authors declare that they have no known competing financial interests or personal relationships that could have appeared to influence the work reported in this paper.

## Data Availability

Deidentified data will be made available upon request to the corresponding author.

## Footnotes

Appendix A Supplementary data associated with this article can be found in the online version at doi:10.1016/j.jhlto.2025.100277.
