# Supplementary material for: Belatacept-based immunosuppression in heart transplant recipients: A single center experience
Source: JHLT Open. 2025 May 22;9:100277. doi: 10.1016/j.jhlto.2025.100277 (PMC12205671; doi:10.1016/j.jhlto.2025.100277)
Supplement: Supplementary file 1 — Supplementary material [file mmc1.docx]

Belatacept-Based Immunosuppression in Heart Transplant Recipients: A Single Center Experience

Wairimu Magua PhD, MS^1^, Maggie Wang MD, MSc^2^, Darlington Pobee MD^2^, Anna Morris PhD^3^, Alexis K. Okoh MD^2^, Emily M. Eichenberger MD, MHS^4^, Geeta M. Karadkhele MS^1^, Divya Gupta MD^2^, J. David Vega MD^1^, Christian P. Larsen MD, PhD^1^, Anne Van Beuningen MD^2^, Alanna A. Morris MD MSc^2^

**Table of Contents**

[**HLA Testing 2**](#_heading=h.niwj5rlr96c)

[**Evaluation of Group Matching 4**](#_heading=h.30j0zll)

[**Actual Tacrolimus Trough Levels 5**](#_heading=h.pmqez3h5euym)

[**Rejection Events During the Follow-Up Period 6**](#_heading=h.1fob9te)

[**Reference 7**](#_heading=h.lcgeinmilqje)

##

## HLA Testing

Mismatches were discerned by evaluating the antigen phenotypes of donors and recipients. Presence of Class I and II HLA antibodies in each patient case were screened using the FlowPRA Class I and II Screening Tests (One Lambda, Inc., Canoga Park, CA). For detection of specific HLA Class I and II antibodies, single antigen bead (SAB) assessment was performed by the LABScreen^TM^ Single Antigen HLA Class I and LABScreen^TM^ Single Antigen HLA Class II kits (One Lambda, Inc/Thermofisher) on the FLEXMAP 3D (Luminex) with the MFI divider off. Sera were pre-treated with fetal calf sera. To circumvent the prozone-like effect, a biotinylated secondary antibody with a 6-atom spacer (Jackson ImmunoResearch) followed with streptavidin-PE was used to detect antibodies bound to LABScreen beads.^1^ For historic cases prior to the routine implementation of SAB testing, FlowPRA was first performed to determine if HLA sensitization was present for Class I or II. If FlowPRA for either Class was greater than 00%, specificity testing was performed by flow cytometric physical crossmatching. For transplants occurring prior to implementation of calculated panel reactive antibodies (cPRA) calculation, we retrospectively calculated cPRA based on HLA specificities assigned positive at the time of transplant using the current cPRA calculator (https://optn.transplant.hrsa.gov/data/allocation-calculators/cpra-calculator/). cPRA was calculated for each case for any specificity identified according to the MFI thresholds for positivity at the time of transplantation, regardless of whether it was made an unacceptable antigen for organ allocation, indicating the overall HLA sensitization in the patient. Following transplantation, recipients were assessed for de novo DSA at 2 weeks and at 1, 2, 3, 6, 12, and 18 months via SAB analysis as stated above.

Flow cytometric crossmatches were performed by incubating recipient sera with pronase-treated surrogate donor peripheral blood lymphocytes. Bound IgG was detected via a secondary FITC-conjugated, Fc-specific, anti-human IgG antibody (Jackson Immunoresearch). PerCP-conjugated monoclonal anti-CD3 and PE-conjugated monoclonal anti-CD19 (BD-Biosciences) were used to simultaneously evaluate T-cell and B-cell populations, respectively. Samples were run and analyzed on the FACSLyric^TM^ (BD Biosciences). Shifts in fluorescence intensity were assessed in ΔMESF (molecules of equivalent soluble fluorochrome) using Quantum™ MESF Kit (Bangs Laboratories). Presumed positive crossmatches were determined by an increase in fluorescence over background of approximately ≥1,200 MESF for T cells and approximately ≥7,000 MESF for B-cells (MESF thresholds might have had slight variations based on negative controls utilized across the transplant era). All sera are run in duplicate along with positive and negative control sera. B cell positive crossmatches were further categorized as attributable to DSA if DSA was present in the recipient. B cell positive crossmatches without presence of DSA are considered false positive crossmatches.

##

##

## Evaluation of Group Matching

**
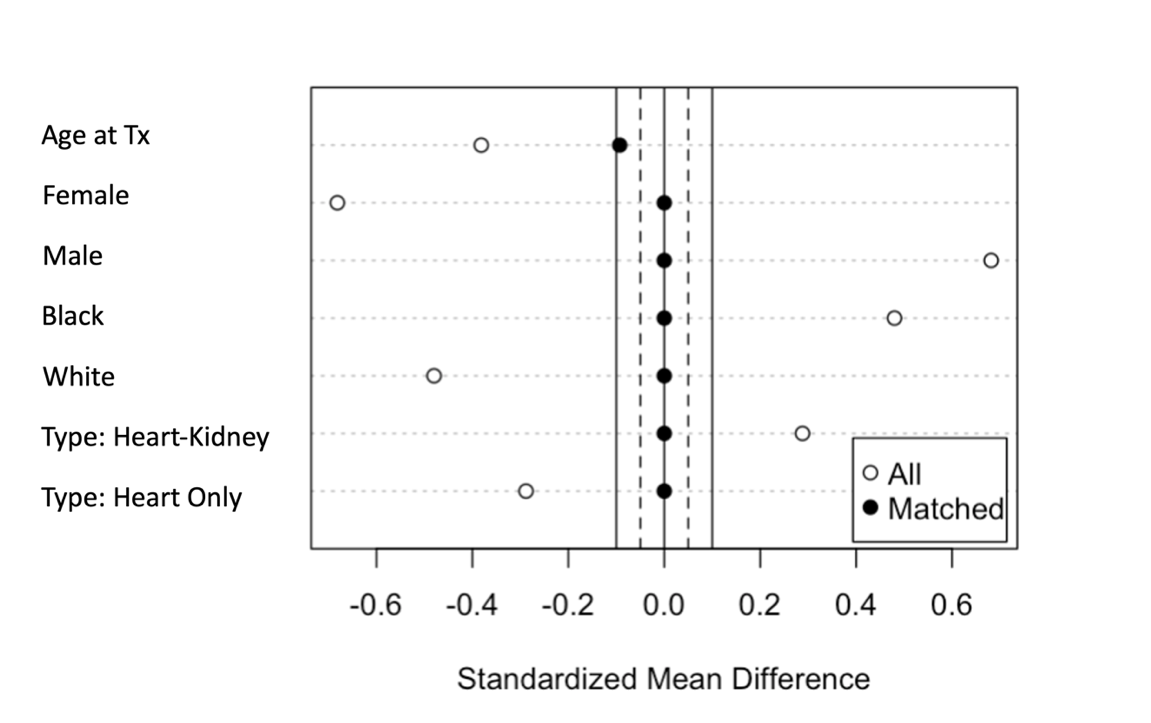
**

**Figure S1**. The mean standardized differences between belatacept and tacrolimus immunosuppression regimen recipient groups were zero by race, gender and type of transplant as they were exact matches*.*

##

## Actual Tacrolimus Trough Levels

**
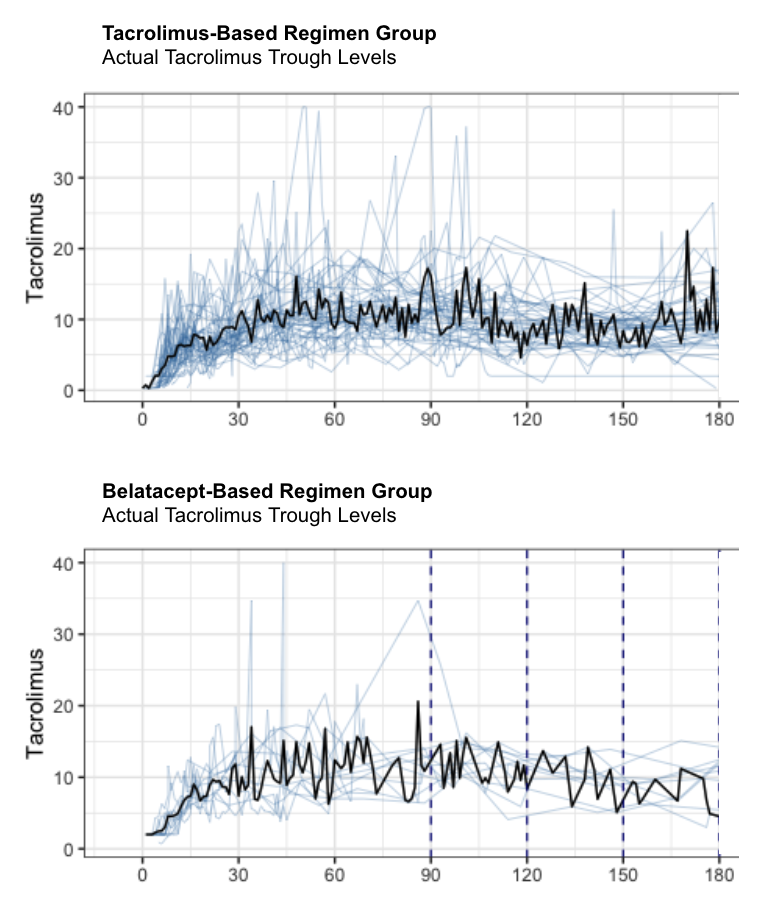
**

**Figure S2.** Median recipient actual trough levels by immunosuppression regimen groups. The vertical lines in the belatacept group represent estimated monthly intravenous infusions days.

## Rejection Events During the Follow-Up Period

**Table S1**. Rejection events during the follow-up period, 86 to 540 days following transplantation. .

| **Rejection**  **Group** | **Tacrolimus**  **Regimen**  N = 48*^1^* | **Belatacept**  **Regimen**  N = 12*^1^* | **p-value***^2^* |
| --- | --- | --- | --- |
| **AMR** |  |  | **>0.9** |
| pAMR0 | 44(92%) | 12 (100%) |  |
| pAMR1 | 3 (6%) | 0 (0%) |  |
| pAMR3 | 1 (2%) | 0 (0%) |  |
| **ACR** |  |  | **0.7** |
| Grade 0R | 42 (88%) | 10 (83%) |  |
| Grade 1R | 6 (13%) | 2 (17%) |  |
| ^1^n (%); ^2^Fisher’s exact test. **AMR:** pAMR0, no histopathologic or immunopathologic features; pAMR1, presence of either histopathologic or immunopathologic features; pAMR3, presence of severe histopathologic and immunopathologic features**. ACR:** Grade 0R (no rejection); Grade 1R (mild), Interstitial and/or perivascular infiltrate with up to 1 focus of myocyte damage. | | | |

##

## Reference

1. Sullivan HC, Gebel HM, Bray RA. Understanding solid-phase HLA antibody assays and the value of MFI. Hum Immunol. Jul-Aug 2017;78(7-8):471-480.
